# Bioclimatic Influence on the Nutritional Composition, In Vitro Ruminal Fermentation Dynamics, and Greenhouse Gas Emissions of *Urtica dioica*

**DOI:** 10.3390/ani15192856

**Published:** 2025-09-30

**Authors:** Khalil Abid, Takwa Abidi, Saifddine Benrajeb, Valentina Balestra, Salvatore Barbera, Rabeb Issaoui, Hatsumi Kaihara, Wijdem Niama, Mohamed Aroua, Mokhtar Mahouachi, Samia Ben Said, Sonia Tassone

**Affiliations:** 1 Department of Agricultural, Forest and Food Sciences, University of Turin, Largo P. Braccini 2, 10095 Grugliasco, Italy; khalil.abid@unito.it (K.A.); salvatore.barbera@unito.it (S.B.); raeb.issaoui@etudiant-issbat.utm.tn (R.I.); hatsumi.kaihara@unito.it (H.K.); 2 Laboratoire d’Appui à la Durabilité des Systèmes de Production au Nord-Ouest, Ecole Supérieure d’Agriculture du Kef, University of Jendouba, Le Kef 7119, Tunisia; abiditakwa506@gmail.com (T.A.); mrsaifddinebenrajeb@gmail.com (S.B.); naaymawejden@gmail.com (W.N.); arouamohamed2310@gmail.com (M.A.); taymallahmah@gmail.com (M.M.); 3 Department of Environment, Land and Infrastructure Engineering, Politecnico di Torino, Corso Duca degli Abruzzi, 24, 10129 Torino, Italy; valentina.balestra@polito.it

**Keywords:** *Urtica dioica*, ruminant nutrition, ruminal fermentation, greenhouse gas emissions, bioclimatic origin, sustainable feed

## Abstract

**Simple Summary:**

Sustainable ruminant production increasingly requires alternative forages that combine high nutritional value with a low environmental footprint. The *Urtica dioica* perennial wild plant is naturally adapted to a wide range of climatic conditions. In this study, *Urtica dioica* was harvested at the early flowering stage from three bioclimatic zones in Tunisia (arid, semi-arid, and sub-humid) and analyzed for nutritional value and greenhouse gas emissions. Across all ecotypes, *Urtica dioica* consistently provided high and stable protein levels. The arid-zone plants contained more structural fiber, polyphenols, and lipids, which reduced digestibility, fermentation efficiency, and metabolizable energy yield, but significantly lowered methane emissions per unit of dry matter, degraded dry matter, and total gas. Semi-arid ecotypes offered a similar nutritional value to sub-humid plants while limiting methane production per unit of dry matter, degraded dry matter, and total gas, representing a balance between productivity and environmental sustainability. These findings demonstrate that both the nutritional quality and enteric methane emissions of *Urtica dioica* are strongly shaped by its bioclimatic origin, with semi-arid ecotypes showing particular promise as climate-resilient, eco-friendly feed resources for ruminant production systems.

**Abstract:**

Climate change, feed shortages, and rising production costs highlight the need for alternative and sustainable forages for ruminants. This study aimed to evaluate the nutritional composition, in vitro ruminal fermentation, and methane emissions of *Urtica dioica* ecotypes originating from contrasting bioclimatic zones in Tunisia. Aerial parts of *Urtica dioica* were harvested at the early flowering stage from arid, semi-arid, and sub-humid regions. Samples were subjected to chemical composition in vitro ruminal fermentation to determine dry matter degradability (DMD), neutral detergent fiber degradability (NDFD), metabolizable energy (ME), and methane production. The results demonstrate that *Urtica dioica* is a promising protein-rich forage, with a stable crude protein content across ecotypes (18.58–20.97% of dry matter). In contrast, NDFD, DMD, ME, and methane emissions varied significantly according to origin. The arid ecotype, characterized by the highest fiber, ether extract, and polyphenol content, exhibited the lowest DMD (53% vs. 61% and 60%), NDFD (45% vs. 55% and 56%), and ME (7.2 vs. 8.6 and 9.0 MJ/kg dry matter) but produced the lowest methane emissions (38.8 vs. 53.2 and 74.2 mL CH_4_/kg DMD) compared with the semi-arid and sub-humid ecotypes. The semi-arid and sub-humid ecotypes had comparable DMD, NDFD, and ME values; however, methane emissions were higher in the sub-humid ecotype. Overall, the semi-arid ecotype provided the most favorable balance between nutritive quality and environmental sustainability. These findings highlight the critical role of ecological origin in determining the feeding value and greenhouse gas footprint of *Urtica dioica*, providing a scientific basis for its potential use as a sustainable forage in ruminant feeding systems.

## 1. Introduction

Ruminant livestock production is increasingly constrained by feed shortages and rising input costs, driven by climate change, land-use competition, and growing animal populations [1,2]. Conventional feeding systems based on cereals and cultivated forages are becoming less sustainable due to their dependence on irrigation, fertilizers, and mechanization [2]. Consequently, there is increasing interest in exploring alternative, underutilized plant species that are naturally adapted to harsh conditions and capable of supporting livestock productivity with reduced environmental and economic costs [2,3]. Among these, *Urtica dioica*, commonly known as stinging nettle, represents a promising candidate. This perennial herbaceous plant of the Urticaceae family is widely distributed and commonly occurs in disturbed or marginal habitats such as roadsides, riverbanks, and fallow land [4,5]. *Urtica dioica* possesses a robust rhizomatous system and several physiological adaptations, such as reduced leaf thickness, enhanced stomatal control, and low epidermal cell density, that allow it to tolerate a wide range of climatic stresses [4,5,6]. Although considered invasive in many agroecosystems [5,7], a recent study has reported that its inclusion in small ruminant diets can improve milk yield, enhance growth performance, and support immune function [8]. Moreover, its use as a partial replacement for conventional forages, such as corn silage or ryegrass silage, has not been associated with negative effects on feed intake, digestibility, or ruminal health [9,10]. Beyond its nutritional value, *Urtica dioica* contains diverse bioactive compounds, including polyphenols, flavonoids, and tannins which confer anti-inflammatory, antihypertensive, diuretic, and anthelmintic properties. This makes it a candidate for integration in phytotherapeutic approaches in veterinary practice [11,12]. Recent meta-analyses have shown that diets enriched with such bioactive substances such as polyphenols, flavonoids, and tannins can beneficially modulate ruminal fermentation by limiting proteolysis and ammonia release, reducing methanogenic archaea, and ultimately lowering the carbon footprint of ruminant production [13,14,15]. Despite its ecological resilience and nutraceutical potential, a critical knowledge gap remains regarding how bioclimatic factors influence the nutritional quality, ruminal fermentability, and greenhouse gas emissions of *Urtica dioica*. This knowledge is essential, as the chemical composition in plants can vary substantially with environmental factors [16]. This study provides the first baseline evaluation of *Urtica dioica* ecotypes, offering fundamental insights into their nutritional composition and ruminal fermentation characteristics to create a foundation for the strategic incorporation of nettle, according to its original ecological origin, into ruminant feeding systems to optimize both nutritional value and environmental sustainability. We hypothesized that *Urtica dioica* ecotypes originating from arid, semi-arid, and sub-humid regions would differ significantly in chemical composition, secondary metabolite content, and ruminal fermentation characteristics. To test this hypothesis, the present study was designed to evaluate the effects of bioclimatic origin on the chemical composition, in vitro ruminal fermentation kinetics, and greenhouse gas emissions of *Urtica dioica*, thereby assessing its suitability as a sustainable and climate-resilient forage resource for ruminant production systems.

## 2. Materials and Methods

### 2.1. Sample Collection

Aerial parts of healthy wild *Urtica dioica* were randomly harvested at the early flowering stage in April 2025 from three ecologically distinct bioclimatic zones in Tunisia: in an arid zone (Sfax governorate; 34.74° N, 10.77° E), a semi-arid zone (Kef governorate; 36.18° N, 8.71° E), and a sub-humid zone (Krib locality, Siliana governorate; 36.34° N, 9.13° E). The classification of these zones was based on the bioclimatic map developed by Chebil et al. [17]. The three bioclimatic zones differed substantially in their environmental parameters (Table 1). All collection sites were characterized by organic soils, contained at least 25% by weight of organic matter, and were free from agrochemical inputs such as fertilizers, pesticides, and herbicides. Approximately 1 kg of fresh biomass from healthy plants was collected per zone. Samples were immediately placed in insulated cool boxes containing ice packs and maintained at approximately 4 °C during transport from the collection sites to the laboratory. Upon arrival, samples were oven-dried at 60 °C for 48 h (Memmert GmbH, Schwabach, Germany), and ground to pass through a 1.0 mm sieve (Retsch GmbH, Haan, Germany). Additionally, a portion of the harvested aerial parts was dried at ambient room temperature, protected from direct sunlight, to preserve the integrity of bioactive compounds for subsequent analysis. Processed samples were stored in airtight, food-grade containers at room temperature (25 ± 2 °C), protected from light and humidity until analysis.

### 2.2. Chemical Composition

All chemical analyses were performed in triplicate. Dry matter (DM) was determined according to method 934.01, crude protein (CP) by the Kjeldahl method (method, 978.04), ether extract (EE) using Soxhlet extraction (method, 920.39), and ash content by incineration at 550 °C (method, 942.05) according to AOAC [19]. Neutral detergent fiber (NDF), acid detergent fiber (ADF), and acid detergent lignin (ADL) were analyzed using the ANKOM 200 Fiber Analyzer (ANKOM Technology, NY, USA), following the Van Soest method [20]. Total polyphenols were quantified using the Folin–Ciocalteu method and expressed as mg of gallic acid equivalents (GAE)/g of DM [21]. Total tannins were determined using the vanillin–HCl assay and expressed as mg of tannic acid equivalents (TAE)/g of DM [22]. Condensed tannins were assessed following Makkar et al. [23], based on the vanillin–sulfuric acid colorimetric reaction, expressed as mg of vanillic acid equivalents (VAE)/g of DM. Non-fibrous carbohydrates (NFCs) were calculated according to the National Research Council equation [24]:

(1) NFC=100−(NDF+CP+EE+ash)

where NFC, NDF, CP, EE, and ash are expressed as % DM.

### 2.3. Ruminal Incubation

#### 2.3.1. Rumen Inoculum Preparation

Rumen fluid was collected weekly from four clinically healthy Piedmontese bulls (aged 16 months) with one bull slaughtered per week over four consecutive weeks at a commercial abattoir in northern Italy, according to Fortina et al. [25]. All animals were raised under identical dietary conditions: 2 kg of barley straw, 4 kg of ryegrass hay, and 10 kg of concentrate per day. Immediately post-slaughter, ruminal content was collected from multiple locations within the rumen, transferred into pre-warmed thermos flasks maintained at 39 °C and delivered to the laboratory within 20 min. The rumen fluid was filtered through a layer of cheesecloth (300 µm porosity) and mixed with a pre-warmed, carbon dioxide (CO_2_)-saturated buffer solution (1:4 *v*/*v*) at 39 °C, following the method of Goering and Van Soest [26].

#### 2.3.2. In Vitro Fermentation

The in vitro fermentation was performed using an automated gas production system (Ritter Apparatebau GmbH & Co. KG, Bochum, Germany) consisting of 18 fermenters, each connected to a dedicated milligas counter, as described by [27]. The system continuously records cumulative gas volume with high temporal resolution, allowing accurate assessment of fermentation kinetics. Meanwhile, all gas produced during the trial was collected in sampling bags.

For each *Urtica dioica* ecotype (*n* = 3), five replicates were prepared, each comprising a total of 2500 mg of DM. Samples were incubated in 500 mL fermentation bottles containing 350 mL of buffered rumen inoculum. Within each replicate, 500 mg of DM was enclosed in a 25 µm porosity filter bag (F57, ANKOM Technology Corp., Macedon, NY, USA), while the remaining 2000 mg of DM was added directly to the fermentation medium. Three additional bottles containing only 350 mL of buffered rumen inoculum and one empty bag were included as blanks. All bottles were sealed and incubated at 39 °C for 48 h under anaerobic conditions.

At the end of incubation, the pH of the fermentation fluid was measured immediately using a pH meter (HALO^®^ model HI11102, Hanna^®^ Instruments, Woonsocket, RI, USA). Gas accumulated in sampling bags was analyzed to determine methane (CH_4_), carbon dioxide (CO_2_), and carbon monoxide (CO) concentrations using portable gas detectors (Dräger X-am 8000 and Dräger X-am 7000, Lübeck, Germany) equipped with a sampling pump [28,29]. Gas volumes for each ecotype were corrected by subtracting the corresponding volumes produced in the blanks. Filter bags were then rinsed in cold water using a turbine washing machine, oven-dried at 60 °C for 48 h, and weighed to determine residue mass. Residues were analyzed for NDF and ADF content using the ANKOM 200 Fiber Analyzer, according to Van Soest et al. [14]. DM degradability (DMD), NDF degradability (NDFD), and ADF degradability (ADFD) were calculated as the percentage of material lost relative to the initial weight, corrected for filter bag weight loss in the blank bottles.

#### 2.3.3. Gas Production Kinetics

Cumulative gas production was modeled using the nonlinear equation of France et al. [30]:

(2) $$Y_{t}=PGP\times (1-e^{\left(-C\times \left(t-Lag\right)\right)})$$

where Y_t_: cumulative gas production at time t (mL/g DM); PGP: potential gas production (mL/g DM), C: fractional rate of gas production (%/hour); and Lag: lag phase before the onset of gas production (hour).

Time to half-maximal gas production (T_1/2_) was calculated as follows:

(3) T12=Lag+ln2C

where T_1/2_: time to half-maximal gas production (hour); C: constant gas production rate (%/hour); and Lag: onset time of gas production (hour).

Average fermentation rate (AFR) was calculated as the average gas production rate up to T_1/2_.

(4) $$\mathrm{AFR}=\frac{PGP\times C}{2(\ln2+C\times Lag)}$$

where AFR: the average fermentation rate (mL/hour); PGP: potential gas production (mL/g DM); C: Constant gas production rate (%/hour); and Lag: onset time of gas production (hour).

#### 2.3.4. Metabolizable Energy and Volatile Fatty Acids

Metabolizable energy (ME) was estimated according to Menke and Steingass [31]:

(5) $$ME=2.20+0.13570\times {GP}_{24}+0.057\times CP+0.0286\times {EE}^{2}$$

where ME: metabolizable energy (MJ/kg DM); GP_24_: net gas production after 24 h (mL/200 mg DM); CP: crude protein content (% DM); and EE: ether extract content (% DM).

Volatile fatty acids (VFA) were estimated according to Getachew et al. [32]:

VFA = 0.0222 × GP_24_ − 0.00425 (6)

where VFA: volatile fatty acids (mmol/200 mg DM) and GP_24_: net gas production after 24 h (mL/200 mg DM).

### 2.4. Statistical Analysis

Gas kinetics and fermentation data were fitted using the nonlinear regression procedure in SAS 9.1 (SAS Institute, Cary, NC, USA). All other parameters were analyzed using the General Linear Model procedure with the following model:

Y_ij_ = μ + α_i_ + ε_ij_ (7)

where Y_ij_: observed value; μ: overall mean; α_i_: fixed effect of the “_i_” bioclimatic zone; and ε_ij_: residual error.

When treatment effects were significant, means were compared using Tukey’s post hoc test where a *p*-value < 0.05 was considered statistically significant.

## 3. Results

### 3.1. Chemical Composition

The chemical composition of *Urtica dioica* varied significantly across bioclimatic zones (Table 2). DM content was highest in the arid ecotype (36.33% of fresh matter), significantly greater than that of the semi-arid (30.34%) and sub-humid (27.26%) ecotypes (*p* < 0.01). NDF content was also highest in the arid ecotype (42.15% DM), while the lowest was recorded in the sub-humid ecotype (29.43% of DM) (*p* < 0.001). ADF values were comparable among all ecotypes, with an average of 22.84% of DM. ADL content was significantly higher in the arid ecotype (6.89% of DM) compared with the other ecotypes (*p* < 0.01). CP ranged from 18.58% to 20.97% of DM, with no significant differences among ecotypes. EE content was significantly higher in the arid ecotype (2.59% of DM), whereas the sub-humid ecotype showed the lowest value (0.63% of DM) (*p* < 0.01). Ash content ranged from 25.10% of DM in the sub-humid ecotype to 28.89% of DM in the semi-arid ecotype, with significant differences detected (*p* < 0.05). NFC increased progressively from the arid (6.62% of DM) to the sub-humid (23.72% of DM) ecotype (*p* < 0.001). Total polyphenol content was highest in the arid ecotype (12.56 mg GAE/g of DM), while total tannins peaked in the semi-arid ecotype (6.37 mg VAE/g of DM). Condensed tannin content did not differ significantly among ecotypes (*p* > 0.05).

### 3.2. Ruminal pH, Degradability, Metabolizable Energy, and Volatile Fatty Acids of Urtica dioica

The ruminal pH, degradability, ME, and VFA concentrations are presented in Table 3. Ruminal pH ranged from 6.12 to 6.19 and was significantly higher in the arid ecotype (*p* < 0.05). DMD was significantly lower in the arid ecotype (52.90%) compared with the semi-arid and sub-humid ecotypes (60.85% and 60.26%, respectively; *p* < 0.01). Similar trends were observed for NDFD, ADFD, ME, and VFA concentrations, all of which were significantly lower in the arid ecotype (*p* < 0.05), with values of 44.83%, 35.34%, 7.16 MJ/kg of DM, and 0.79 mmol/200 mg of DM, respectively. By contrast, the semi-arid and sub-humid ecotypes showed higher average values of 55.75%, 49.01%, 8.78 MJ/kg of DM, and 1.05 mmol/200 mg of DM, respectively.

### 3.3. Gas Production Kinetics of Urtica dioica

**Figure 1:**
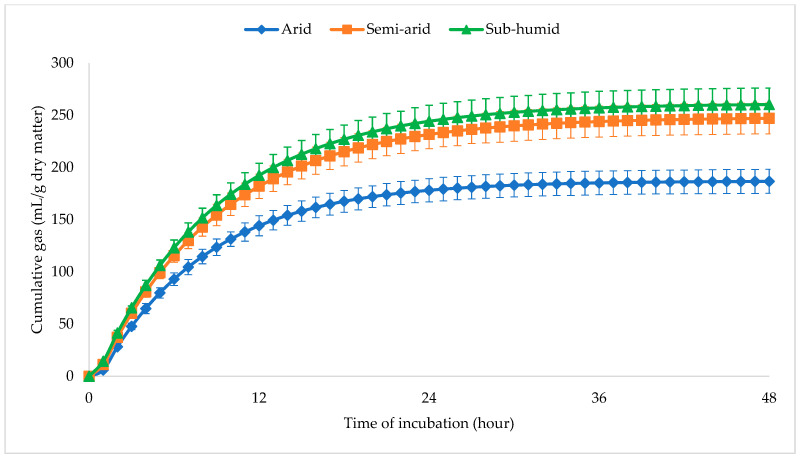
Bioclimatic influence on the cumulative gas production kinetics of *Urtica dioica.*

Gas production kinetics differed significantly among ecotypes (Table 4; Figure 1). The arid ecotype had the lowest potential gas production (187 mL/g DM), the fastest gas production rate constant, the longest Lag time, and the lowest AFR. No significant difference was noted between the gas kinetics of semi-arid and sub-humid ecotypes.

### 3.4. Greenhouse Gas Emissions of Urtica dioica

Greenhouse gas emissions during fermentation are reported in Table 5. CH_4_ production was significantly lower in the arid ecotype, both in absolute terms and when expressed as a percentage of total gas or per unit of DMD. The highest CH_4_ emissions were observed in the sub-humid ecotype, both in absolute terms, when expressed as a percentage of total gas or per unit of DMD. CO_2_ production was similar in semi-arid and arid ecotypes both in absolute terms and when expressed as a percentage of total gas or per unit of DMD and lower than sub-humid ecotype. CO was significantly higher in the arid ecotype, both in absolute terms and when expressed as a percentage of total gas or per unit of DMD.

## 4. Discussion

### 4.1. Chemical Composition of Urtica dioica

The chemical composition of *Urtica dioica* was markedly influenced by bioclimatic origin. Plants collected from the arid zone exhibited significantly higher NDF and ADL levels, reflecting increased lignification as a plant response to water stress, likely aimed at minimizing transportational loss through enhanced cell wall thickness [16]. Temperature probably also affects these parameters, since lower temperature can reduce the ability of plants to lignify their secondary cell walls [33], and the rise in temperature increases NDF content [34]. Compared with conventional forages used in ruminant nutrition, the NDF content of *Urtica dioica* from the arid region was comparable to that of alfalfa harvested at mid-bloom (43.9% of DM), whereas plants from semi-arid and sub-humid regions exhibited NDF levels similar to alfalfa harvested at early bloom (33.1% of DM) [35]. CP levels remained stable across ecotypes (18.6–21.0% of DM), comparable to traditional protein forages used in ruminant nutrition, such as alfalfa in bloom stage (18–21% of DM) [35] and better than main roughages commonly used in Tunisia, such as oat hay (8.0% of DM) [36] and wheat straw (3.2% of DM) [37]. These high CP content and stability highlight *Urtica dioica* as a potentially reliable protein source across varying environments with proper diet control in ash inclusion. EE content varied with bioclimatic conditions, being highest in the arid ecotype (2.59% of DM) and lowest in the sub-humid ecotype (0.63% of DM). This range is typical for herbaceous forages and remains well below fat levels in total diets considered detrimental to rumen function (≈>6% of DM) [38]. The higher EE in the arid ecotype may reflect increased cuticular waxes and membrane lipids under drought, as previously reported [39,40]. Ash content was also influenced by bioclimatic conditions, which significantly lower the values in the sub-humid environment. This reduction may be due to greater biomass accumulation of plants under favorable conditions [41], yet the values still exceeded the recommended safe limits for ruminants (12–14% DM) [42], suggesting potential issues with palatability or mineral imbalance [42]. Our results indicate a slightly higher ash content compared with previous reports, in which minerals accounted for approximately 20% of dry matter in *Urtica dioica* [43,44], yet lower than the values reported by Arros et al. [45], who observed ash contents reaching 29% DM. This elevated mineral content in our study may be explained by the harvesting period, as collection in April has been associated with higher concentrations of minerals in the leaves of this species [46]. NFC content increased progressively from arid to sub-humid ecotypes, likely due to the improved photosynthetic activity and carbohydrate accumulation under less stressful conditions [47,48]. Polyphenol and tannin contents also varied significantly. Total polyphenols were highest in the arid ecotype, likely due to drought-induced oxidative stress activating phenylpropanoid pathway [49]. Compared with conventional forages, the total polyphenol content of *Urtica dioica* exceeded that of alfalfa (5.65 mg GAE/g of DM) and ryegrass (8.41 mg GAE/g of DM) [50]. However, total tannins displayed a nonlinear response to climate stress, with a peak in the semi-arid ecotype, possibly due to severe drought conditions limiting tannin synthesis [51]. By contrast, condensed tannins were stable across all ecotypes and consistently higher than those reported for conventional forages such as alfalfa (0.12 mg CE/g of DM) and ryegrass (0.19 mg CE/g of DM) [50].

### 4.2. Ruminal Fermentation Dynamics and Degradability of Urtica dioica

Despite the growing interest in unconventional forages, data on ruminal fermentation dynamics and degradability of *Urtica dioica* remain scarce. Our study showed that ruminal fermentation parameters, ruminal degradability, ME, and VFA production, were similar in ecotypes from semi-arid and sub-humid environments but significantly higher than those from arid environments. These differences can be attributed to several unfavorable chemical traits of the arid ecotype. The elevated ADL content acts as a physical and chemical barrier, limiting microbial colonization of plant tissues and restricting enzymatic access to structural carbohydrates and other content, thereby reducing ruminal fermentation and degradability [52]. In addition, the higher EE content of the arid ecotype can hinder microbial adhesion to feed particles and exert inhibitory effects on microbial growth and activity [38,53]. Furthermore, the elevated polyphenolic content negatively affects ruminal fermentation by forming complexes with proteins and carbohydrates, inhibiting microbial enzymatic activity, and ultimately reducing digestibility and energy yield [54]. Comparison with previous studies from other geographical origins, Purcell et al. [55] reported a VFA production of 0.92 mmol/200 mg of DM in Irish ecotypes harvested in the spring, which was higher than that observed for arid ecotype but lower than the VFA values recorded for our semi-arid and sub-humid ecotypes. The more intense fermentation activity observed in semi-arid and sub-humid ecotypes resulted in slightly lower ruminal pH compared with those from arid regions. However, all ecotypes maintained levels within the optimal physiological range for rumen function from 6.0 to 7.0 [56], indicating adequate buffering capacity and microbial stability during fermentation of this species. *Urtica dioica* from semi-arid and sub-humid environments exhibited DMD and GP values comparable to those of alfalfa (DMD: 65.2–66.2%; GP: 201–213 mL/g of DM), a widely used protein-rich forage in ruminant nutrition [35]. Moreover, these ecotypes showed even higher NDFD than alfalfa (NDFD: 40.3–40.9%) [35]. In contrast, the arid ecotype showed similar NDFD to alfalfa, but had markedly lower DMD and GP values [35]. These findings highlight the nutritional relevance of *Urtica dioica* as a promising alternative protein-rich forage, particularly suitable for ruminant feeding systems in semi-arid and sub-humid regions. In comparison with previous studies from other geographical origin, Kulivand and Kafilzadeh [57] reported a GP of 223 mL/g of DM for Iranian ecotypes, which was higher than that observed in our arid ecotype but lower than the GP values recorded for our semi-arid and sub-humid ecotypes. Purcell et al. [55] reported DMD of 81% in Irish ecotypes harvested in the spring, substantially higher than the DMD measured across all ecotypes in our study. underscoring the rumen fermentability of this plant is highly context dependent. Although the arid ecotype is nutritionally less suitable due to a lower ruminal fermentation, degradability, and ME, it was characterized by significantly lower enteric CH_4_ emissions whether expressed per unit of DM, DMD, or of total gas. This reduction is likely attributable to the higher lipid content in the arid ecotype [58], as lipids enhance biohydrogenation pathways that compete with methanogenesis by redirecting metabolic hydrogen (H_2_) away from CH_4_ synthesis. In addition, its higher polyphenol content likely suppressed methanogenic archaea by impairing their enzymatic activity and disrupting hydrogenotrophic methanogenesis [58]. The CH_4_ values recorded in the arid ecotype were markedly lower than those typically reported for conventional protein-rich forages such as alfalfa, which produces between 38 and 48 mL CH_4_/g of DM and 59 and 69 mL CH_4_/g of DMD under in vitro fermentation conditions [35]. This positions the arid ecotype as a strategic forage in climate-smart ruminant feeding programs. Interestingly, while the arid ecotype had reduced degradability and energy yield, it was also associated with the highest CO concentrations and CO_2_ levels, comparable to those of the semi-arid ecotype. This paradox can be explained by the suppression of methanogenic archaea due to high polyphenol concentrations. These compounds disrupt coenzyme functions and inhibit key steps in the methanogenesis pathway, reducing CH_4_ synthesis [59]. As a result, less hydrogen is converted into methane, leading to increased accumulation of CO or CO_2_ during ruminal fermentation [29].

Although the semi-arid and sub-humid ecotypes showed similar ruminal degradability and fermentability, significant differences were observed in their CH_4_ emissions. The sub-humid ecotype produced substantially higher levels of CH_4_, even exceeding the values typically reported for alfalfa (59–69 mL CH_4_/g of DMD) during in vitro fermentation [35]. This elevated CH_4_ output is likely linked to a lower concentration of fermentation-inhibiting compounds, particularly lipids and polyphenols such as tannins, creating a more favorable environment for the growth and activity of methanogenic archaea [58,59]. By contrast, the semi-arid ecotype represents an optimal compromise between nutritive value and environmental impact. It maintained GP, DMD, ME, and VFA levels comparable to the sub-humid ecotype, while maintaining CH_4_ emissions within the conventional range reported for alfalfa [35]. This favorable balance between nutritional value and environmental footprint positions the semi-arid ecotype as a particularly promising candidate for inclusion in sustainable ruminant feeding strategies, especially in agroecosystems where both productivity and climate resilience are key priorities. Comparison with previous studies from other geographical origins Purcell et al. [55] reported a CH_4_ output of 17.4% of total gas for Irish ecotypes harvested in spring, comparable to the values recorded for our sub-humid ecotype. By contrast, Kulivand and Kafilzadeh [57] reported higher CH_4_ production (21.4% of total gas) in Iranian ecotypes, which exceeds the levels observed in our study in all ecotype. This discrepancy may be explained by differences in the vegetative stage at harvest, as the Iranian ecotypes were collected at the mid-vegetative stage, while our ecotypes were harvested during early flowering.

## 5. Conclusions

This study highlights *Urtica dioica* as a promising protein-rich forage with stable CP content across ecotypes. Nevertheless, other nutritional components, ruminal fermentability, degradability, ME, VFA, and greenhouse gas emissions were strongly influenced by ecological origin. The arid ecotype was characterized by the highest NDF, ADL, EE, and polyphenol contents, exhibited the lowest ruminal fermentation, degradability, ME, VFA, but produced lower CH_4_ emissions, both in absolute terms and when expressed as a percentage of total gas or per unit of degraded DMD. In contrast, the sub-humid and semi-arid ecotypes showed comparable ruminal fermentation, degradability, ME, and VFA although CH_4_ emissions were significantly higher in the sub-humid ecotype both in absolute terms and relative to total gas or DMD. Overall, the semi-arid ecotype offered the most favorable balance between nutritional quality and environmental sustainability. These findings underscore the critical role of bioclimatic origin in determining the feeding value and environmental footprint of *Urtica dioica*. Future research should build on these baseline insights by exploring the implications of bioclimatic origin of *Urtica dioica* on ruminant health and productive performance, with particular emphasis on its integration into practical feeding system.

## Figures and Tables

**Table 1:** Monthly average temperature (°C), precipitation (mm), relative humidity (%), and sunshine (hour) in the study zones.

	Temperature			Precipitation			Humidity			Sunshine		
	Arid	Semi-Arid	Sub-Humid	Arid	Semi-Arid	Sub-Humid	Arid	Semi-Arid	Sub-Humid	Arid	Semi-Arid	Sub-Humid
January	13	9	9	9	22	22	67	69	74	230	218	218
February	14	11	10	17	27	30	66	68	73	278	269	265
March	16	13	12	22	60	68	64	64	72	338	320	322
April	18	16	15	14	54	54	61	59	71	349	336	337
May	21	20	20	5	37	34	57	53	67	388	381	387
Juin	25	26	25	1	17	19	53	47	62	390	385	386
July	28	29	29	0	4	4	50	43	59	406	406	411
August	29	29	29	10	19	21	50	44	58	381	376	379
September	27	25	24	21	42	46	60	56	66	336	328	328
October	23	19	18	27	41	42	64	63	71	321	312	311
November	18	14	14	27	37	36	64	64	70	277	264	265
December	14	10	11	9	36	38	67	69	74	279	271	270

Nomad season [18].

**Table 2:** Bioclimatic influence on the chemical composition of *Urtica dioica.*

	Arid	Semi-Arid	Sub-Humid	SEM	*p*-Value
DM	36.33 ^a^	30.34 ^b^	27.26 ^b^	2.921	**
NDF	42.15 ^a^	33.25 ^b^	29.43 ^b^	3.413	***
ADF	23.77	22.21	22.55	2.312	NS
ADL	6.89 ^a^	5.79 ^b^	5.76 ^b^	0.231	**
CP	20.67	18.58	20.97	1.221	NS
EE	2.59 ^a^	1.66 ^b^	0.63 ^c^	0.243	**
Ash	27.97 ^a^	28.89 ^a^	25.10 ^b^	2.011	*
NFC	6.62 ^c^	17.62 ^b^	23.72 ^a^	3.321	***
Total polyphenol	12.56 ^a^	9.48 ^b^	10.54 ^b^	1.021	*
Total tannin	2.88 ^b^	6.37 ^a^	3.30 ^b^	1.020	*
Condensed tannin	0.46	0.37	0.59	0.341	NS

Different superscripts (a, b, c) within a row indicate statistically significant differences (*p* < 0.05) between bioclimatic zones; NS: *p*-value ≥ 0.05 (not significant); *: *p* < 0.05; **: *p* < 0.01; ***: *p* < 0.001; DM: dry matter (% fresh matter); NDF: neutral detergent fiber (% dry matter); ADF: acid detergent fiber (% dry matter); ADL: acid detergent lignin (% dry matter); CP: crude protein (% dry matter); EE: ether extract (% dry matter); ash (% dry matter); NFC: non-fiber carbohydrates (% dry matter); total polyphenol (mg gallic acid equivalents/g dry matter); total tannin (mg tannic acid/g dry matter); condensed tannin (mg vanillic acid equivalents/g dry matter); and SEM: standard error of the mean.

**Table 3:** Bioclimatic influence on ruminal fermentation, ruminal degradability, metabolizable energy, and volatile fatty acids of *Urtica dioica.*

	Arid	Semi-Arid	Sub-Humid	SEM	*p*-Value
Ph	6.19 ^a^	6.13 ^b^	6.12 ^b^	0.030	*
DMD	52.90 ^b^	60.85 ^a^	60.26 ^a^	4.331	**
NDFD	44.83 ^b^	55.48 ^a^	56.01 ^a^	5.222	**
ADFD	35.34 ^b^	48.17 ^a^	49.85 ^a^	4.169	**
ME	7.16 ^b^	8.60 ^a^	8.96 ^a^	0.671	**
VFA	0.79 ^b^	1.02 ^a^	1.08 ^a^	0.061	**

Different superscripts (a, b) within a row indicate statistically significant differences (*p* < 0.05) between bioclimatic zones; *: *p* < 0.05; **: *p* < 0.01; DMD: dry matter degradability (%); NDFD: neutral detergent fiber degradability (%); ADFD: acid detergent fiber degradability (%); ME: metabolizable energy (MJ/kg dry matter); VFA: volatile fatty acids (mmol/200 mg dry matter); and SEM: standard error of the mean.

**Table 4:** Bioclimatic influence on gas production kinetics of *Urtica dioica.*

	Arid	Semi-Arid	Sub-Humid	SEM	*p*-Value
PGP	187 ^b^	248 ^a^	261.3 ^a^	17.32	***
C	0.131 ^a^	0.116 ^b^	0.116 ^b^	0.1091	**
Lag	0.75 ^c^	0.59 ^a^	0.52 ^a^	0.212	*
T_1/2_	6.86	6.57	6.48	0.351	NS
AFR	15.95 ^b^	18.84 ^a^	20.13 ^a^	1.412	*

Different superscripts (a, b, c) within a row indicate statistically significant differences (*p* < 0.05) between bioclimatic zones; NS: *p*-value ≥ 0.05 (not significant); *: *p* < 0.05; **: *p* < 0.01; ***: *p* < 0.001; PGP: potential gas production (mL/g dry matter); C: fractional rate of gas production (%/hour); Lag: lag phase before the onset of gas production (hour); T_1/2_: time to half-maximal gas production (hour); AFR: average fermentation rate (mL/hour); and SEM: standard error of the mean.

**Table 5:** Bioclimatic influence on greenhouse gas emissions of *Urtica dioica* under in vitro ruminal fermentation.

	Arid	Semi-Arid	Sub-Humid	SEM	*p*-Value
Proportion of total gas (%)					
CH_4_	10.98 ^c^	13.21 ^b^	17.60 ^a^	1.220	***
CO_2_	38.74 ^ab^	32.60 ^b^	44.15 ^a^	4.231	*
CO	0.082 ^a^	0.052 ^b^	0.055 ^b^	0.0132	**
Emission per dry matter (mL/g DM)					
CH_4_	20.45 ^c^	32.36 ^b^	44.70 ^a^	2.239	**
CO_2_	72.45 ^b^	79.87 ^b^	112.10 ^a^	9.441	**
CO	0.155 ^a^	0.128 ^b^	0.140 ^ab^	0.0291	*
Emission per dry matter degradability (mL/g DMD)					
CH_4_	38.82 ^c^	53.17 ^b^	74.18 ^a^	3.222	***
CO_2_	136.95 ^b^	131.25 ^b^	186.20 ^a^	17.231	***
CO	0.293 ^a^	0.210 ^b^	0.223 ^b^	0.0451	*

Different superscripts (a, b, c) within a row indicate statistically significant differences (*p* < 0.05) between bioclimatic zones; *: *p* < 0.05; **: *p* < 0.01; ***: *p* < 0.001; DM: dry matter; DMD: dry matter degradability; and SEM: standard error of the mean.

## Data Availability

The data presented in this study are available on request from the corresponding author.

## Abbreviations

ADF	Acid detergent fiber
ADFD	Acid detergent fiber degradability
ADL	Acid detergent lignin
AFR	Average fermentation rate
C	Fractional rate of gas production
CH_4_	Methane
CO	Marbon monoxide
CO_2_	Carbon dioxide
CP	Crude protein
DM	Dry matter
DMD	Dry matter degradability
EE	Ether extract
Lag	Lag phase before the onset of gas production
ME	Metabolizable energy
NDFD	Neutral detergent fiber degradability
NFC	Non-fibrous carbohydrates
PGP	Potential gas production
SEM	Standard error of the mean
T_1/2_	Time to half-maximal gas production
TAE	Tannic acid equivalents
VAE	Vanillic acid equivalents
VFA	Volatile fatty acids
